# Effectiveness of Low-Intensity Extracorporeal Shock Wave Therapy in Erectile Dysfunction: An Analysis of Sexual Function and Penile Hardness at Erection: An Umbrella Review

**DOI:** 10.3390/jpm14020177

**Published:** 2024-02-04

**Authors:** Esther M Medrano-Sánchez, Belén Peña-Cantonero, Paloma Candón-Ballester, María Blanco-Díaz, Esther Díaz-Mohedo

**Affiliations:** 1 Research Group CTS305, Department of Physical Therapy, Faculty of Nursing, Physical Therapy and Podiatry, Universidad de Sevilla, 6, Avenzoar St., 41009 Sevilla, Spain; emedrano@us.es; 2 CitizenFisio, Carmona Rd., 41008 Seville, Spain; belenpenacantonero@gmail.com; 3 Neus Gramage, Asunción St., 41011 Seville, Spain; pcandonballester@gmail.com; 4 Physiotherapy and Translational Research Group (FINTRA-RG), Institute of Health Research of the Principality of Asturias (ISPA), Faculty of Medicine and Health Sciences, Universidad de Oviedo, 33006 Oviedo, Spain; 5 Department of Physical Therapy, Universidad de Málaga, Francisco Peñalosa Av., 29071 Málaga, Spain; estherdiaz@uma.es

**Keywords:** extracorporeal shock wave therapy, erectile dysfunction, physical therapy

## Abstract

The present umbrella review of five systematic reviews and meta-analyses was conducted to investigate the effectiveness of Low-Intensity Extracorporeal Shock Wave Therapy (Li-ESWT) in the treatment of vascular origin Erectile Dysfunction (ED). A search was carried out in the databases of Pubmed, Scopus, Medline, Scielo and Embase. Participants were divided into two groups: an experimental group receiving Li-ESWT and a control group receiving simulated shock waves. The main variable of this study is ED, measured using the International Index of Erectile Function-Erectile Function (IIEF-EF) and the Erection Hardness Score (EHS) scale. The results showed a statistically significant increase in the mean IIEF-EF score in the experimental group. Overall, four out of five articles reported an increase in the EHS score in the Li-ESWT group compared to the placebo. Concerning the treatment parameters, better outcomes were observed with an energy density of 0.09 mJ/mm^2^ and the application of 1500–2000 pulses. Additionally, a follow-up of 6–12 months resulted in greater improvement in ED compared to 3 months, although more studies investigating follow-ups beyond 12 months are needed. Obtaining conclusive and clear results is challenging; however, everything indicates that Li-ESWT is an innovative therapeutic alternative for vascular-origin ED due to its low risk and improvement in erectile function.

## 1. Introduction

Erectile dysfunction (ED) is the inability to achieve or maintain an erection sufficient for satisfactory sexual performance [1]. Erectile dysfunction (ED) is considered a major health problem when it persists for at least three months and can be caused by organic (vascular, neurological, hormonal, anatomical, drug-induced), psychological or a combination of both [1,2].

It is estimated that one in five men aged 40 to 80 [3,4] have ED, and its incidence is likely to increase over time. Despite being a common sexual dysfunction, it is often undiagnosed, as patients rarely report their symptoms to healthcare professionals [5].

**Figure 1:**
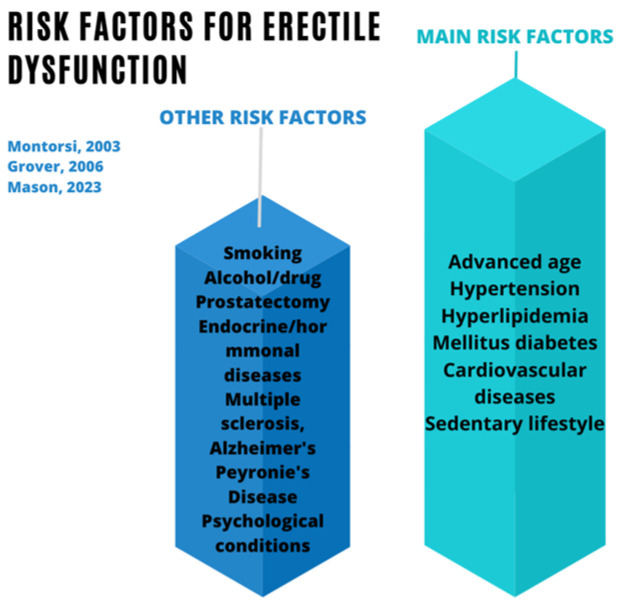
Risk factors for erectile dysfunction [6,7,8].

For many years, it has been assumed that ED is a common process of aging, in which many men normalize their loss of sexual function [5]. However, apart from advanced age, obesity, hypertension, hyperlipidemia, diabetes mellitus, and cardiovascular diseases are the main risk factors for the development of ED [6,7,8]. These risk factors, along with other factors associated with the onset of ED described by other authors, can be found in Figure 1 [6,7,8,9].

A study by Hodges et al. [10] found that men with ED are more likely to have a cardiovascular event within 5 years of diagnosis [6]. This suggests that ED may be a sign of underlying cardiovascular disease. For this reason, men with ED should be evaluated for cardiovascular risk factors and monitored for early signs of heart disease [11,12,13].

Although ED is not a pathology in itself, it has a significant impact on the quality of life of men who experience it, as well as their partners and families, reaching to affect their level of self-esteem, emotional management, and relationship problems. It has been demonstrated that satisfaction with sexual life is an important predictor of satisfaction with life in general, therefore, men with ED who cannot have satisfying sexual relations will have their overall quality of life diminished [12,13,14].

The first-line treatment [2] for ED consists of changes in lifestyle, modification of risk factors, and pharmacotherapy with phosphodiesterase type 5 inhibitors (PDE5i), including sildenafil (Viagra), tadalafil, and vardenafil [15]. These drugs are not initiators of erection by themselves and require sexual stimulation for it to occur. Their effects produce sufficient rigidity of the penis for vaginal penetration; however, despite having high rates of efficacy and safety, they are limited to treating the symptoms of ED without correcting the underlying pathophysiology, such as structural, neurological, or vascular lesions secondary to other pathologies that are responsible for ED [16]. In addition, some patients do not respond to treatment, have it contraindicated, or present various adverse effects such as headaches, nasal congestion, or dizziness [15]. In these cases, other alternative therapeutic options can be used, such as intraurethral and intracavernosal alprostadil applications, injections of stem cells and platelet-rich plasma (PRP), vacuum pump devices, and surgically implanted penile prostheses [17,18]. In addition, cognitive-behavioral therapy and couple-directed therapy can help improve ED when it has a psychological component [2].

Currently, there is research studying the treatment of ED with pelvic floor exercises [19] and manometric biofeedback [20,21]. Dorey et al. [22] studied the effectiveness of pelvic floor exercises combined with the use of manometric biofeedback along with recommendations for lifestyle changes in patients with ED compared to another group that was only advised on modifications of their habits. The results showed that, at 6 months of treatment with active pelvic floor work and biofeedback, more than half of the patients in the study improved.

In the last decade, numerous studies have been conducted on the use of low-intensity extracorporeal shock wave therapy (Li-ESWT) in patients with ED. The main objective of this technique is to act on the pathophysiology of ED in order to prevent the deterioration of erectile function [13]. The mechanism of action of the shock waves is that they, when interacting with the target tissues, trigger a series of biological reactions that release growth factors and that, in turn, stimulate neoangiogenesis to promote neovascularization [23,24,25]. In addition, Li-ESWT is considered a safe and non-invasive method that causes minimal side effects [26,27].

In this way, Vardi et al. [28] first evaluated the efficacy of Li-ESWT for ED. This pilot study included 20 men with vasculogenic ED who had previously responded to PDE5. The treatment protocol consisted of applying Li-ESWT to the corpus and pillars of the penis in two weekly sessions for 3 weeks, which were repeated after a 3-week interval without treatment. After 1 month, significant increases were recorded in the domain scores of the International Index of Erectile Function-Erectile Function (IIEF-EF) in all men, as well as an improvement in erection duration, rigidity, and penile endothelial function. This type of intervention did not produce pain or adverse events during follow-up. The results of the studies seem to be encouraging, showing improvements in both IIEF-EF and Erection Hardness Score (EHS) after the use of Li-ESWT [16].

The main objective of our study is to collect the published scientific evidence in systematic reviews with meta-analyses on the effectiveness of Li-ESWT in ED and to determine whether it is effective in improving sexual function and penile hardness at erection.

## 2. Materials and Methods

### 2.1. Design

The present study constitutes an umbrella review of systematic reviews accompanied by meta-analyses. It has been conducted following the guidelines outlined in the PRISMA statement [29], which is essential for the study’s proper integrity.

In this umbrella review, an analysis of the existing scientific literature on the effects of Li-ESWT in patients with vascular-origin ED and how it can help improve sexual function and penile hardness at erection is performed. The exploration was carried out in electronic databases, including Pubmed, Scopus, Embase, Scielo, and Medline. For this study, research conducted between the year 2013 (inclusive) and June 2023 has been selected.

The registration number for this review has been requested from the National Institute for Health Research (PROSPERO).

### 2.2. PICO Question

To formulate the PICO question, a population of men with vascular-origin ED treated with Li-ESWT and compared to a placebo group has been considered. The expected outcomes are focused on the improvement of ED. Thus, the research question has been formulated as follows: Is low-intensity extracorporeal shockwave therapy effective in men with vascular-origin erectile dysfunction for improving sexual function and penile hardness at erection?

### 2.3. Eligibility Criteria

Regarding the articles selected for this review, a series of inclusion and exclusion criteria were considered before conducting the study.

Inclusion criteria that had to be met to accept studies in this review were:

Research that comprises systematic reviews and meta-analyses.

### 2.4. Information Sources

The electronic databases used for the literature searches were Pubmed, Scopus, Embase, Scielo, and Medline. These searches were conducted between March (inclusive) and June 2023.

### 2.5. Search Strategy

The initial search in these databases has allowed us to explore the existing research described in the current scientific literature and gain a comprehensive understanding of the results we can expect, based on other studies to support future conclusions.

The keywords used during the process were the following Medical Subject Headings (MeSH): Extracorporeal Shockwave Therapy and Erectile Dysfunction. The Boolean operator “AND” was used between these terms.

The search strategy used in the Pubmed database was (“Extracorporeal Shockwave Therapy” [Mesh]) AND “Erectile Dysfunction” [Mesh], applying the filters “human”, “systematic review”, and “meta-analysis”.

In Scopus, the search strategy was formulated as follows: (“shockwave” AND “therapy” AND “erectile” AND “dysfunction”). The filter used was “review”.

In Embase, the combination of descriptors used was (“extracorporeal shockwave therapy” AND “erectile dysfunction”), applying the filters “human”, “systematic review”, and “meta-analysis”.

The search strategy used in Scielo involved the terms (“extracorporeal shockwave therapy” AND “erectile dysfunction”), with no filters applied.

In Medline, the search strategy was (“erectile dysfunction” AND “extracorporeal shockwave therapy”), with no filters applied.

### 2.6. Study Selection Process

This study was carried out through the consensus of three evaluators. After conducting the search in the databases mentioned earlier, a study selection process was performed by screening those that met the previously established eligibility criteria. By reading the title and abstract of the results, studies that addressed the treatment of vasculogenic ED with Li-ESWT were selected, particularly systematic reviews accompanied by meta-analyses.

### 2.7. Data Extraction Process

Two authors were responsible for data extraction by selecting the studies and identifying their characteristics (participants, interventions, and outcomes) through reading the full-text papers.

### 2.8. Study Variable

The variable in this study is Erectile Dysfunction (ED), an ordinal qualitative variable that has been measured in all the studies included in the research using the International Index of Erectile Function-Erectile Function (IIEF-EF) questionnaire and the Erection Hardness Score (EHS).

The IIEF-EF is a validated, multidimensional, and self-administered questionnaire considered the gold standard for evaluating male sexual function [30]. This questionnaire consists of 15 questions and should be completed by individuals who are sexually active (engaging in sexual activity with a partner or manual stimulation). Scores range from 0 to 5 for questions 1 to 10 and from 1 to 5 for questions 11 to 15, which examine the five major domains of male sexual function: erectile function, orgasmic function, sexual desire, satisfaction with sexual intercourse, and overall satisfaction. The final score allows categorizing ED as severe, moderate, mild, or no ED. One of the main benefits of this scale is its ability to provide a clear and objective diagnosis of ED, determining its severity [31,32]. This questionnaire, along with scoring details, can be found in the “Supplementary Materials” section (Figure S1 and Table S1).

On the other hand, the EHS is a single-item patient-reported outcome for rating erection hardness. This scale is a simple, reliable, and valid tool for assessing erection hardness in clinical trial research. The score ranges from 0 (the penis does not enlarge), 1 (the penis enlarges but is not hard), 2 (the penis is hard enough for penetration but not completely hard), 3 (the penis is hard enough for penetration but not completely rigid) to 4 (the penis is completely hard and fully rigid). Although it does not assess other factors related to ED, the EHS allows patients to score the hardness of their erection in a straightforward manner, which provides valuable information to healthcare professionals [30,33].

### 2.9. Methodological Quality Assessment

The evaluation of the methodological quality and risk of bias in the studies included in this review was conducted using the AMSTAR tool [34], a validated instrument supported by reproducible evidence for assessing the internal validity of systematic literature reviews. It consists of 16 items that globally assess the methodological quality of a systematic review, determining whether it is of low, moderate, or high quality. Two independent evaluators participated in this assessment, with the involvement of a third evaluator anticipated to resolve any potential discrepancies between the two main evaluators.

## 3. Results

### 3.1. Study Selection

Following the literature searches in the aforementioned databases, the study selection process was carried out, as described in a flowchart (Figure S1. Initially, a total of 911 references were identified, and after removing duplicate documents, the titles and abstracts of 517 studies were reviewed, of which 508 were excluded for not meeting the eligibility criteria. In Pubmed, a total of 102 records were found, and seven were selected for full-text reading [1,4,16,25,27,35,36]. In Scopus, 246 studies were identified, and six were selected [1,3,16,25,35,37], of which four were duplicated in Pubmed, so only two of them were included for full-text reading [3,37]. In Embase, a total of 560 studies were found, and seven were selected for full-text reading. However, all of them were duplicates from Pubmed and Scopus, so no study from Embase was selected for full-text reading.

In total, nine studies [1,3,4,16,25,27,35,36,37] were selected for evaluation in full-text, and after reading them, only five were included in this review [4,25,27,35,37]. The excluded studies included four, with two of them not using the 15-question IIEF-EF scale [1,3], and the other two not addressing vasculogenic ED [16,36].

Furthermore, studies were identified through other search methods. One study [38] was selected through citation searching, but after reading it in full, it was excluded for not addressing vasculogenic ED.

The total number of clinical trials included in the five meta-analyses selected for this umbrella review was 25, of which 12 appeared in more than one meta-analysis. The number of overlapped RCTs are provided in Table 1.

### 3.2. Evaluation of the Quality of the Selected Studies

The methodological quality of the studies included in this review was assessed by two reviewers using the AMSTAR scale. Each reviewer independently analyzed each study according to the criteria of the aforementioned scale. The scale consists of 16 questions, the answers to which determine the methodological quality of the studies. After evaluating each study, the reviewers shared the following results (Table 2):

As can be seen, there were differences in the results of three of the five studies analyzed, so the participation of a third reviewer was necessary to resolve the discrepancies generated between the two main reviewers. After the intervention of this third reviewer, the final results were as follows (Table 3):

Therefore, it can be concluded that the studies selected for this review have a medium-high methodological quality.

### 3.3. Characteristics of the Selected Studies

The following are the main characteristics of the studies selected for this review, as detailed in Table S1: author and year of publication, number of RCTs and total number of participants, intervention groups, energy density and number of Li-ESWT pulses, treatment duration, measurements taken, treatment follow-up, and meta-analysis results.

### 3.4. Synthesis of the Results

The IIEF-EF was the evaluation tool used in all the meta-analyses and systematic reviews studied [4,25,27,35,37], with the aim of observing the efficacy of Li-ESWT for ED. On the other hand, the EHS was also evaluated by the five studies; however, only four of them showed a quantitative analysis of this tool [4,25,35,37]. Other aspects evaluated were: Sexual Encounter Profile (SEP), energy flux density (EFD), number of shockwave pulses, treatment duration and safety, patient satisfaction, sexual health inventory for men (SHIM), global assessment questionnaire (GAQ) and maximum systolic velocity (PSV); however, they were only taken into account in some articles.

#### 3.4.1. Synthesis of the Results for Erectile Function

Yao et al. [4] conducted follow-ups at 1, 3 and 6 months, and found that the Li-ESWT group significantly increased the IIEF-EF scores compared to the placebo group. Furthermore, three types of ED were described according to severity, taking into account the baseline IIEF-EF, which were: severe group (baseline IIEF-EF < 12), moderate group (IIEF-EF = 12–17) and mild group (baseline IIEF-EF > 17), concluding that the improvement in IIEF-EF in the treatment group was greater than that in the control group, regardless of the subgroup.

The group of Dong et al. [25] studied erectile function at 1, 3, 6, 9 and 12 months. The results obtained demonstrated improvements from the beginning of treatment compared to placebo. Furthermore, a statistically significant improvement in the IIEF-EF score was observed in patients with both moderate and severe ED.

Angulo et al. [27] observed that Li-ESWT in patients with ED was effective in the short term, since IIEF-EF increased significantly during the first month of treatment, both in the experimental group and in the control group. Despite this, the improvement of the Li-ESWT group was maintained over time. The efficacy of placebo was significantly greater at the beginning of treatment; however, there are insufficient data to assess the long-term response. Furthermore, the active treatment group showed significant improvements at the 3 to 6 month follow-up.

The review with meta-analysis carried out by Liu et al. [35] carried out a study of the results at 7 weeks and 1, 3, 6, 9 and 12 months, after which an evidently high improvement in the IIEF-EF score was observed in patients belonging to the experimental group compared to with those in the control group, both in mild, moderate and severe ED.

Finally, Sokolakis et al. [37] obtained a significantly elevated difference in IIEF-EF in the experimental group compared to the placebo group from the beginning of treatment, with a follow-up of 1, 3, 6 and 12 months. Likewise, the percentage of patients who achieved a minimal clinically important difference (MCID) in the IIEF-EF score corresponds to the Li-ESWT group. Additionally, during this study, the use of PDE5i was prohibited during treatment, so a washout period of 2 to 4 weeks was performed for patients taking this medication. In this way, participants were grouped into two subgroups: patients who responded and those who did not respond to PDE5i before starting treatment. The results showed that there were no significant differences in the IIEF-EF score during the follow-up months in the group of patients who responded to the drug; however, there were differences with respect to baseline IIEF-EF.

#### 3.4.2. Synthesis of the Results for the Erection Hardness Score

The group of Yao et al. [4] showed a significant difference in the EHS of the number of patients in the Li-ESWT group regarding the control group, with the baseline score being less than or equal to 2, and obtaining a score greater than or equal to 3 after treatment. The study by Dong et al. [25] observed a significant increase in EHS during the first month of treatment, regardless of the number of shock waves used. Liu et al. [35] established evidently high EHS improvements in the three ED subgroups (severe, moderate and mild), regardless of the number of shock waves applied. Finally, Sokolakis et al. [37] presented results showing an EHS greater than or equal to 3 during follow-up in a higher percentage of patients in the Li-ESWT group compared to the control group. Furthermore, it was observed that men who responded to PDE5i showed a score greater than or equal to 3 during follow-up in the active treatment group.

Overall, the results obtained show that EHS was markedly improved in ED patients who underwent Li-ESWT compared to those in the placebo group.

#### 3.4.3. Synthesis of the Results for the Sexual Encounter Profile

The research carried out by Yao et al. [4], used questions SEP2 and SEP3 as additional evaluation criteria, obtaining a result that was not statistically significant for the Li-ESWT group compared to the control group. In the research of Sokolakis et al. [37], SEP2 and SEP3 were also evaluated in some studies; however, they were not used consistently in all investigations and their subsequent evaluation was not carried out.

#### 3.4.4. Synthesis of the Results for the Energy Flux Density

In the study by Yao et al. [4] two subgroups were established to evaluate the IIEF-EF. The subgroup that included the studies that worked with an EFD of 0.09 mJ/mm^2^ and the subgroup that included those that used 0.1 and 0.2 mJ/mm^2^. The results established that erectile function improved significantly in both subgroups. In the research of Dong et al. [25] studies used an EFD of 0.09 mJ/mm^2^ and 0.15 mJ/mm^2^ with a frequency of 5 Hz. The studies evaluated by Angulo et al. [27] and Liu et al. [35] used an EFD of 0.09 mJ/mm^2^ and 0.25 mJ/mm^2^. In the study by Sokolakis et al. [37], the EFD used by research ranged between 0.05 mJ/mm^2^ and 0.25 mJ/mm^2^, although most studies used 0.09 mJ/mm^2^, obtaining a significant difference in erectile function in the Li-ESWT group.

#### 3.4.5. Synthesis of the Results for the Number of Pulses

To investigate the number of pulses, Yao et al. [4] carried out a subdivision into 3 groups. The first group used 600 pulses, the second between 1500 and 2000 pulses, and the number of pulses used by the third group was greater than 3000. The results established that the first group did not obtain a significant improvement in the IIEF-EF, despite to show encouraging results; however, groups 2 and 3 significantly improved erectile function. In the research of Dong et al. [25], some studies applied a total of 18,000 shocks (1500 pulses/session), another study 15,000 (3000 pulses/session) and another study 3000 (600 pulses/session); however, the latter reported a worsening of the effect of the treatment, due to the application of a smaller number of shocks. On the other hand, the studies evaluated by Angulo et al. [27] were divided into three subgroups that used between 14,400 and 20,000 pulses, 18,000 pulses and 36,000 pulses. Liu et al. [35] showed that their investigations applied 15,000 and 18,000 pulses. Finally, the studies evaluated by Sokolakis et al. [37] applied shocks ranging between 600 and 5000 pulses, although most studies administered 1500 pulses/treatment (a total of 3000–60,000 pulses), which produced a statistically significant improvement in IIEF-EF.

#### 3.4.6. Synthesis of Results for the Duration of Treatment

Sokolakis et al. [37] was the only study that studied the duration of treatment, establishing that the total number of sessions influences the effectiveness of the treatment.

#### 3.4.7. Synthesis of the Results for the Safety of the Treatment

Angulo et al. [27] and Sokolakis et al. [37] were the only studies that evaluated the safety of the treatment. In the first meta-analysis with a systematic review [27], the majority of patients did not present adverse effects; however, among those who presented it, there were mild adverse effects, such as a slight allergic reaction to the gel or pain in the penis. In the case of the second investigation [37], only some local inflammation was reported in one of their studies and one patient who was diagnosed with Peyronie’s disease 6 months after shock wave treatment.

#### 3.4.8. Synthesis of Results for Patient Satisfaction

Angulo et al. [27] evaluated patient satisfaction one month after treatment, resulting in 75–80% of patients considering the treatment effective and recommended. Furthermore, after one year, 61% of patients felt satisfied or very satisfied with the therapy.

#### 3.4.9. Synthesis of the Results for Maximum Systolic Velocity

PSV was studied only by Sokolakis et al. [37]. Data on penile hemodynamics were collected at baseline and during follow-up, obtaining a significant improvement in PSV from baseline compared to the control group and reaching higher levels during follow-up. No data were obtained on the percentage of patients who did not improve or worsened in the control and treatment groups; however, PSV was reported to be increased in all but one patient.

## 4. Discussion

Shock wave therapy has been used in various medical fields to treat different conditions such as tendinopathies [51], osteoarthritis [52], kidney stones [53], and Peyronie’s Disease [54], among others. In recent years, numerous studies have emerged evaluating the efficacy of Li-ESWT in vascular-origin ED. The first randomized, double-blind, sham-controlled study was conducted in 2012 by the team of Vardi et al. [45], which showed promising results on the short-term clinical and physiological efficacy of Li-ESWT in erectile function in men with ED responsive to PDE5i. Since then, numerous studies have been conducted to assess shock waves in patients with different types and severity of ED, using different devices and parameters, with the aim of evaluating their effects on these patients and, if useful, determining which yields better outcomes.

The present study is an umbrella review of five systematic reviews and meta-analyses that encompass a total of 56 RCTs and 3.908 patients with ED. Its purpose is to compare the efficacy of Li-ESWT to that of a placebo in the treatment of vascular-origin ED, with the goal of evaluating erectile function and penile hardness at erection using the IIEF-EF and EHS, respectively. Additionally, other aspects, such as SEP, EFD, number of pulses, treatment duration, safety, patient satisfaction, and PSV, were also investigated.

In all five included studies, it was observed that, within a timeframe of 1 to 12 months, the improvement in erectile function was greater in the Li-ESWT group compared to the control (placebo) group from the outset of each study, as assessed using the validated IIEF-EF questionnaire. Thus, subjective results suggest that shock waves could enhance erectile function in patients with ED, regardless of the severity of the dysfunction. Penile hardness at erection was also examined in all five studies included; however, conclusive results were only obtained in four of them, as Angulo et al. [27] did not have sufficient data to conduct the corresponding statistical analysis. The validated EHS questionnaire was used to assess penile hardness, demonstrating a significant subjective improvement in the questionnaire score in the experimental group compared to the control group, regardless of the number of shock waves applied. These findings appear to indicate that shock waves are also effective in enhancing penile hardness at erection and are promising, as they may enhance the quality of sexual life for men who suffer from this condition.

Furthermore, PSV exhibited a significant increase from its baseline value in the experimental group, indicating that the hemodynamic function of the penis, following treatment application, provides objective data showing improvement in ED through Li-ESWT. These results suggest that Li-ESWT may enhance erectile function both subjectively (validated questionnaires) and objectively (penile hemodynamics) in patients with vasculogenic ED.

In general, the results of the analyzed studies indicate that patients with ED who received Li-ESWT significantly improved their IIEF-EF and EHS scores compared to those who received placebo treatment. Specifically, in Dong et al.’s study [25], the IIEF-EF score increased by up to five points after the application of Li-ESWT compared to the initial score.

The improvement in IIEF-EF scores in patients with varying degrees of ED severity seems to differ. It has been observed that this improvement is more pronounced in patients with moderate ED than in men with mild or severe ED [4]. However, the data obtained are not sufficient to draw conclusions regarding the effectiveness of Li-ESWT based on the baseline severity of ED. Additionally, the improvement in IIEF-EF also varies depending on the treatment duration and follow-up time. Dong et al. [25] found that treatments lasting less than 6 weeks showed a significant increase in IIEF-EF compared to 9-week treatments. In this case, the treatment protocol used (EFD and the number of shockwaves) must be considered to provide a coherent explanation for the significant improvement in IIEF-EF in treatments lasting less than 6 weeks. Initially, it has been observed that improvement after 6 months of follow-up is greater than after 1 and 3 months [4]. This could be explained by the therapeutic effect of Li-ESWT being directly proportional to the follow-up time, meaning the results suggest that the benefits of this therapy are maintained in the short-to-medium term. However, further research is needed to study the long-term effects of Li-ESWT for at least 12 months of follow-up. On the other hand, the studies by Angulo et al. [27] and Liu et al. [35] are notable for recording short-term improvements in baseline IIEF-EF scores in the control (placebo) group, but none of them were clinically significant. In other words, there are no existing data to demonstrate whether the improvement achieved by the placebo is sustained over time.

On the other hand, there is a wide range of research utilizing treatment protocols with different EFD and pulse numbers. In the present umbrella review, it has been observed that Li-ESWT can have varying effects on erectile function depending on the EFD used or the pulses applied. In the majority of studies analyzed in this umbrella review, it has been confirmed that an EFD of 0.09 mJ/mm^2^ yields a greater improvement in IIEF-EF compared to an EFD of 0.1–0.25 mJ/mm^2^ [4,25,35,37]. Regarding the pulses applied, it has been noted that a greater number of shock waves is associated with a significant increase in IIEF-EF [4,16,25,27,35], in contrast to other studies that administered fewer shock waves; and a lower number of pulses can even lead to a worsening of the Li-ESWT effect [25]. For example, Yao et al. [4] state that between 1500 and 2000 shock waves per treatment provide more benefits than 600 or 3000 discharges; however, Dong et al. [25] report heterogeneous results in terms of the number of pulses applied in each study, which could be explained by the different treatment protocols adopted in each investigation. Regarding the duration of treatment, some heterogeneity is also detected between the results. For instance, Dong et al. [25] establishes that treatments lasting less than 6 weeks yield a greater therapeutic effect than 9 weeks of duration, and Sokolakis et al. [37] suggest that re-treating patients 6 months after the treatment may improve erectile function without producing side effects; however, these results did not reach statistical significance. Therefore, it could be established that lower energy density (0.09 mJ/mm^2^), a greater number of pulses, and shorter treatments (<6 weeks) lead to greater therapeutic efficacy [4,25,27,35].

Current research on Li-ESWT indicates that it is a non-invasive, safe, and low-risk treatment with mild adverse effects, as compared to second or third-line treatments for ED. Furthermore, it has been observed that patients who do not respond to PDE5i treatment can also benefit from the therapeutic effect of Li-ESWT [4]. Angulo et al. [27] assessed patient satisfaction after one month of treatment, resulting in 75–80% of patients considering the treatment effective and recommendable. Additionally, after one year, 61% of patients reported feeling satisfied or very satisfied with the therapy. Although data on patient satisfaction are limited, research on this aspect suggests that a significant percentage of men with ED are satisfied with Li-ESWT treatment and would recommend it to other men in the same situation [27]. However, the results obtained in the present study do not provide sufficiently objective data to draw significant conclusions.

In the current scientific literature on this study topic, quite heterogeneous results can be found regarding the treatment protocols used for ED. Considering the results extracted from the present umbrella review and comparing them with the conclusions drawn by other lines of research, some differences can be observed. A study conducted in 2017 by the group of Zou et al. [38] observed that a 9-week treatment protocol with an energy density of 0.09 mJ/mm^2^ and 1.500 pulses achieved a better therapeutic effect than a 5-week protocol, both in terms of IIEF and EHS. The treatment’s effectiveness in terms of erectile function was 8.31 times higher in the Li-ESWT group than in the control group. Regarding penile hardness, the improvement was 2.50 times greater in the experimental group. The study also evaluated whether risk factors for ED, such as age or different diseases like diabetes or cardiac conditions, influenced the results obtained, concluding that studies with participants who had more severe risk factors had worse outcomes than studies with participants who had milder risk factors. However, these conclusions did not reach statistical significance. The results extracted from this research are similar to those of the present study; however, although in most of their studies, ED was of vascular origin, in one of them, ED was caused by nerve-sparing prostatectomy. Additionally, it was observed that patients who did not respond to PDE5i treatment were more likely to obtain favorable results; however, no significant conclusions were obtained either.

Currently, most of the results seem to indicate that Li-WEST is effective in patients who respond to PDE5i; however, for men who do not respond to pharmacological treatment, only one study conducted by the group of Kitrey et al. [43] managed to demonstrate with significant results that Li-WEST could also improve erectile function in patients who do not respond to PDE5i.

The study by Sokolakis et al. [37] is worth noting, in which there are sufficient data indicating that Li-ESWT could improve the erectile function of men with vascular-origin ED who respond to PDE5i, with at least a clinically important difference (MCID) in the IIEF-EF score (>4 points). The Minimum Clinically Important Difference (MCID) is considered an ideal method to assess the actual clinical effectiveness of interventions. The MCID in the IIEF-EF score has been determined to be 4 points, indicating that a 4-point difference can be clinically significant for patients with ED. For this reason, the use of MCID has been recommended as a precise and meaningful tool for evaluating the effectiveness of Li-WEST treatment in the future [55]. The results from Sokolakis and his group suggest that patients who do not respond to PDE5i have a lower response rate than those who respond to PDE5i or do not receive treatment with this drug, which may be related to a higher prevalence of moderate or severe ED [37]. Furthermore, another study conducted in 2018 by the group of Kalyvianakis et al. [56] demonstrated that patients who responded better to Li-ESWT were likely to be younger and even had favorable results with pharmacological treatment; however, the results regarding treatment effectiveness were independent of the initial severity of the disease. Therefore, further studies are needed to determine the response rate of Li-WEST, taking into account the initial severity of the disease and the optimal treatment protocol for each case. This heterogeneity in the studies leads to a wide range of results, making it more difficult to establish a clear conclusion about the efficacy of Li-ESWT in ED.

On the other hand, in 2016, Clavijo et al. [3] demonstrated that erectile function showed a statistically significant improvement from the beginning of the follow-up in men with ED compared to those subjected to sham therapy. However, this study used a shortened erectile function scale, using the 6-item form, IIEF-6. When analyzing the data from this research, compared to the other reviews and meta-analyses included in the umbrella review, there is a greater incongruity of erectile function data, as the scale’s abbreviation does not allow for the most homogeneous comparison. Therefore, due to the inclusion of a heterogeneous population of patients with ED (caused by vascular and non-vascular factors, responders and non-responders to PDE5i, etc.), a definitive conclusion regarding the ideal treatment for each population has not been reached.

In relation to the mechanism of action of shockwaves on the pathogenic function of the penis in ED, it is not clear [36]. However, it has been observed that low-energy shockwaves could promote blood flow and stimulate the release of vascular growth factors that induce angiogenesis, such as vascular endothelial growth factor (VEGF) [57]. This could explain why Li-ESWT is being studied in cases of vascular-origin ED and not in other conditions such as Peyronie’s disease or neurogenic ED. Additionally, flow-mediated dilation has significantly improved in the conducted research, indicating improvements in both endothelial function and penile hemodynamics [42,44]. Fode et al. [58] observed in various animal models that the improvement in erectile function could be due to the stimulation of mechanosensors, thus inducing the improvement of microcirculation, nerve regeneration, remodeling of erectile tissue with an increase in the muscle-collagen ratio, and the reduction of inflammatory processes and cellular responses to stress. Furthermore, in 2019, Sokolakis and his team conducted research to study the erectile tissue of naturally aging rats and observe their response to shockwave therapy. It was observed that Li-ESWT could reduce sympathetic nervous system activity [59]. These data seem to indicate that the main advantage of the treatment is the potential to restore natural erectile function; however, most of these studies show preliminary results, with no significant answers regarding the actual mechanism of action of Li-ESWT.

Some of the limitations observed in this research were that the MCID was not analyzed in all studies. Therefore, despite obtaining conclusive and significant results about the effectiveness of Li-WEST in ED, the improvement in the IIEF-EF score in most studies is less than 4 points, which may mean that these studies do not have sufficient clinical value, as the MCID is considered an essential tool as a standard for evaluation. Another significant limitation was found in the initial search, in which there were investigations with a wide range of populations, studying Li-ESWT treatment in patients with ED of various origins, chronic pelvic pain, Peyronie’s disease, or even ED following radical prostatectomy. This leads to a more heterogeneous population, further complicating the development of an ideal treatment protocol for ED. Furthermore, the variables studied (IIEF-EF, EHS, SEP, etc.) are not analyzed by all studies, and the results are generally examined between 1 and 12 months, without the ability to draw relevant long-term conclusions about Li-ESWT treatment. On the other hand, factors such as age, diabetes, hypertension, coronary diseases, or hyperlipidemia are elements that influence the severity of ED and, therefore, the therapeutic effect of shockwaves; however, relevant conclusions about the factors related to ED and how they influence treatment response have not yet been obtained. Moreover, the variability found in studies, in which participants are on adjunct treatments such as PDE5i during Li-ESWT application, does not allow for conclusive results regarding whether pharmacological treatment is useful or not when combined with shockwaves to improve erectile function, although some research seems to indicate that it may be effective.

Different Li-ESWT protocols should be investigated with as homogeneous populations as possible to identify the optimal EFD, the ideal number of sessions, considering the session interval and frequency, and the total number of shockwaves applied. In addition, the inclusion of a population taking any other treatment that may affect erectile function during the study should be limited to avoid confusion. More well-designed RCTs with follow-ups exceeding 1 year are needed in the future to compare the effectiveness of Li-ESWT alone vs. in combination with PDE5i for vascular-origin ED and to determine if one provides greater benefits than the other. It would also be highly beneficial for other healthcare professionals, especially physicians (urologists and cardiologists), to consider this treatment option for ED, include it in first-line conservative treatment, and recommend it to patients who may benefit from it, avoiding, as much as possible, more invasive treatments.

Due to the wide methodological repertoire of the studies, it is challenging to obtain conclusive and clear results. However, despite the non-uniform protocols, devices, energy density parameters, and Li-ESWT doses, all studies obtained encouraging results. These findings seem to indicate that Li-ESWT has positive effects on vascular-origin ED.

## 5. Conclusions

This umbrella review of five systematic reviews and meta-analyses concludes that Li-ESWT, in comparison to placebo, may improve erectile function by significantly increasing IIEF and EHS scores in patients with mild-to-moderate vasculogenic ED. However, shock wave generating devices, setting parameters, treatment duration, and patient characteristics are also important factors influencing the effectiveness of Li-ESWT, and furthermore, its specific mechanism of action remains unknown. Therefore, well-designed, controlled, more homogeneous research with extended treatment follow-up is needed to demonstrate, with a high level of evidence, the mechanisms of action of Li-ESWT in ED that support this conclusion. Everything indicates that Li-ESWT is a novel therapeutic alternative for vascular-origin ED, given its low risk, minimal side effects, and improvement in erectile function for men who have difficulty achieving and maintaining an erection during sexual intercourse.

## Figures and Tables

**Table 1:** RCTs repeated in two or more meta-analyses (MT) included in the umbrella review.

RCTs Repeated in Two or More Meta-Analyses (MT) Included in the Umbrella Review	Yao et al. [4]	Dong et al. [25]	Angulo et al. [27]	Liu et al. [35]	Sokolakis et al. [37]
Fojecky et al. [39]	x	x		x	x
Kalyvianakis and Hatzichristou [40]	x	x		x	x
Olsen et al. [41]	x	x	x	x	x
Kim et al. [42]	x			x	
Kitrey et al. [43]	x	x		x	x
Srini et al. [44] (2015)	x	x	x	x	x
Vardi et al. [45]	x	x	x	x	x
Sramkova et al. [46] (2020)	x				x
Yamaçake et al. [47] (2019)	x			x	x
Vinay et al. [48] (2021)	x			x	
Yee et al. [49] (2014)	x	x	x	x	x
Zewin et al. [50] (2018)	x				x

**Table 2:** Results of the quality assessment by two reviewers.

	Reviewer 1	Reviewer 2
Yao et al. [4]	Yes (12)	Yes (12)
	Partial yes (0)	Partial yes (0)
	No (4)	No (4)
Dong et al. [25]	Yes (11)	Yes (13)
	Partial yes (1)	Partial yes (1)
	No (4)	No (2)
Angulo et al. [27]	Yes (9)	Yes (8)
	Partial yes (1)	Partial yes (0)
	No (6)	No (8)
Liu et al. [35]	Yes (11)	Yes (11)
	Partial yes (1)	Partial yes (1)
	No (4)	No (4)
Sokolakis et al. [37]	Yes (12)	Yes (13)
	Partial yes (0)	Partial yes (0)
	No (4)	No (3)

**Table 3:** Final results of the quality assessment using the AMSTAR scale.

	Reviewer 1 + Reviewer 2 + Reviewer 3
Yao et al. [4]	Yes (12)
	Partial yes (0)
	No (4)
Dong et al. [25]	Yes (13)
	Partial yes (1)
	No (2)
Angulo et al. [27]	Yes (9)
	Partial yes (1)
	No (6)
Liu et al. [35]	Yes (12)
	Partial yes (1)
	No (3)
Sokolakis et al. [37]	Yes (13)
	Partial yes (0)
	No (3)

## Data Availability

The data presented in this study are available in Figure S1 and Table S1.

## Supplementary Materials

The following supporting information can be downloaded at: https://www.mdpi.com/article/10.3390/jpm14020177/s1, Figure S1: Risk factors for erectile dysfunction; Table S1: Characteristics of the Selected Studies.
