# Supplementary material for: Effectiveness of Low-Intensity Extracorporeal Shock Wave Therapy in Erectile Dysfunction: An Analysis of Sexual Function and Penile Hardness at Erection: An Umbrella Review
Source: J Pers Med. 2024 Feb 4;14(2):177. doi: 10.3390/jpm14020177 (PMC10890328; doi:10.3390/jpm14020177)
Supplement: Supplementary file 1 [file jpm-14-00177-s001.zip › jpm-2806408-supplementary.pdf]

Supplementary A

Figure S1. Risk factors for erectile dysfunction

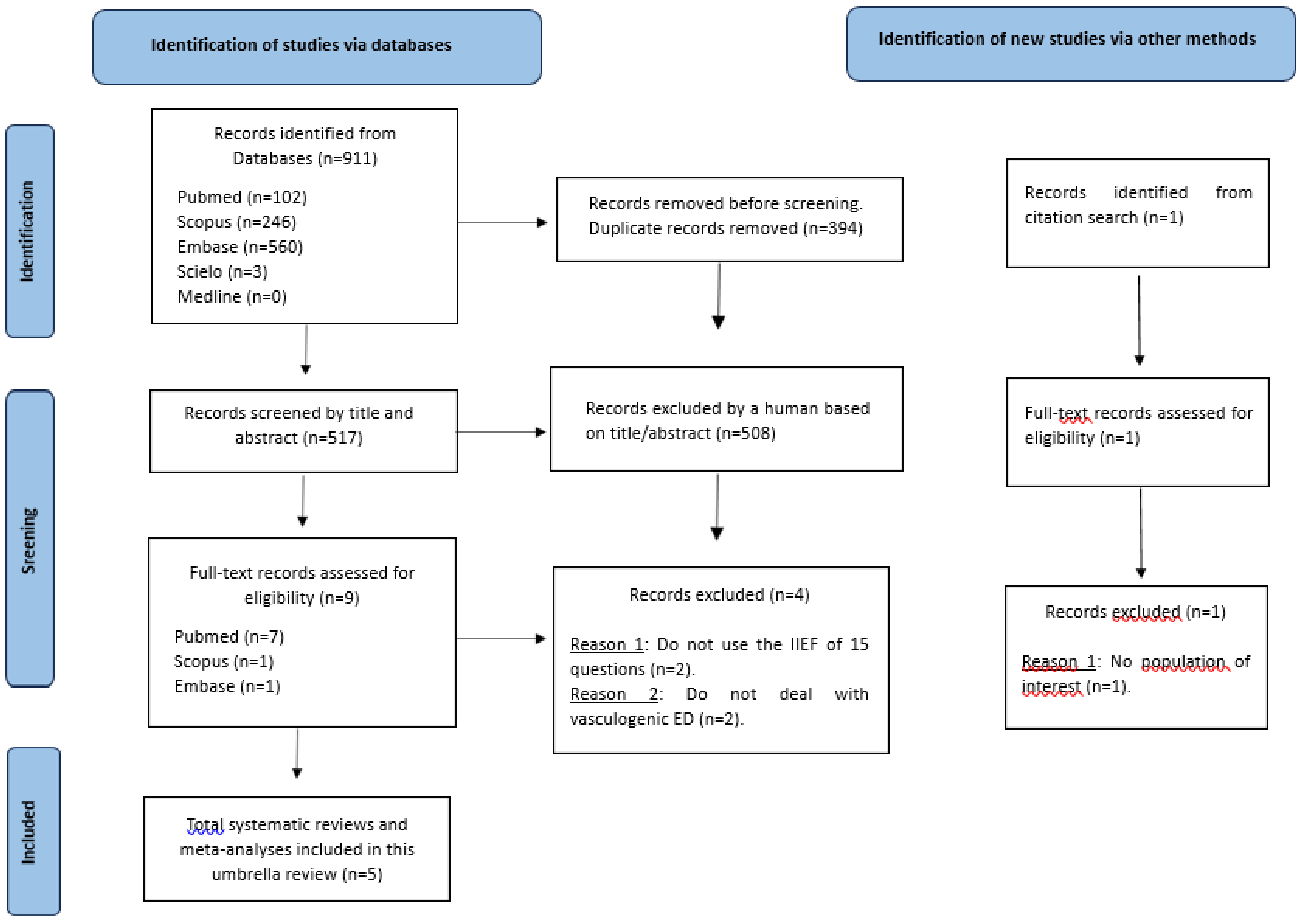

Supplementary B

Table S1. Main characteristics of the studies included in this umbrella review. Source: Own elaboration.

**Author and year of publication.**	**Number of RCT and total number of participants.**	**Intervention groups.**	**Energy density and number of pulses of Li-ESWT.**	**Treatment application period.**	**Measurements made.**	**Treatment monitoring.**	**Meta-analysis results.**
**Yao et al. 2022 [4]**	16 RCT and 1.064 participants.	- **Experimental group:** Li-ESWT.<br>- **Control group:** Placebo using simulated wave.	- **Groups according to energy density (14/16 RCT):** 0.09 mJ/mm^2 and 0.1-0.2 mJ/mm^2.<br>- **Groups by number of pulses per treatment (14/16 RCT):** 600, 1.500-2.000, and > 3.000 shock wave pulses.	- **10/16 RCT:** 2 times/week for 3 weeks + 3 weeks without treatment + 3 weeks of treatment (8 RCT), 3 weeks of treatment (1 RCT), or 4 weeks of treatment (1 RCT).<br><br>- **6/16 RCT:** 1 time/week for 4 weeks (2 RCT), 5 weeks (3 RCT), or 8 weeks (RCT) of treatment.	**- Erectile function** (IIEF-EF).<br>- **Erection hardness score** (EHS).<br>- **Sexual encounter profile** (SEP).	1, 3 and 6 months.	- **IIEF-EF:** Significant improvement after 1 month (MD = 3.18, 95% CI = [1.38, 4.98], *p* = 0.0005), 3 months (MD = 3.01, 95% CI = [2.04, 3.98], *p* < 0.00001), and 6 months (MD = 3.20, 95% CI = [2.49, 3.92], *p* < 0.00001) of treatment in the experimental group compared to the control group.<br>- **EHS:** 8/16 RCT: Initial EHS ≤ 2 → EHS ≥ 3 after treatment. Significant improvement in EHS in the experimental group: OR = 5.07, 95% CI = [1.78, 14.44], *p* = 0.002.<br>- **SEP:** Not statistically significant: SEP2: OR = 1.27, 95% CI = [0.70, 2.30], *p* = 0.43; SEP3: OR = 4.24, 95% CI = [0.67, 26.83], *p* = 0.13.
**Dong et al. 2019 [25]**	7 RCT and 522 participants.	- **Experimental group:** Li-ESWT.<br>- **Control group:** Simulated	- **Groups by energy density:** 0.09 mJ/mm^2 → 6/7 RCT.	**- 5/7 RCT:** 2 times/week for 3 weeks + 3 weeks without treatment + same treatment for another 3 weeks.	**- Erectile function** (IIEF-EF).<br>- **Erection hardness score** (EHS).	1, 3, 6, 9 and 12 months.	- **IIEF-EF:** Significant improvement after 1 month (MD = 1.19 points, 95% CI = [0.30, 2.09], I^2 =77%, *p* = 0.009) of treatment in the experimental group compared to the control group. Significant improvement in

		shock wave with probes.	0.15 mJ/mm^2 → 1/7 RCT. Frequency = 5 Hz.<br>- **Groups by number of pulses per treatment:** 3.000, 15.000, and 18.000 discharges.	**- 1/7 RCT:** 1 time/week for 5 weeks + 5 weeks without treatment.<br>**- 1/7 RCT:** 5 times/week for 4 weeks + 1 week without treatment + same treatment for another 4 weeks.			pooled mean IIEF-EF scores from baseline to follow-up (3,6,9 and 12 months) (MD = 1.99 points, 95% CI = [1.35, 2.63], I^2 = 64%, p < 0.00001) compared to simulated therapy. Significant increase in the change in IIEF-EF score in the experimental group compared to the control group (MD = 3.62 points, 95% CI = [2.99, 4.25], I^2 =51%, p < 0.00001).<br><br>- **EHS:** 3/7 RCT: Significant improvement after 1 month of Li-ESWT (OR: 16.02; 95% CI [7.93, 32.37]; I^2 = 38%, p < 0.00001).
**Angulo et al. 2017 [27]**	12 RCT and 636 participants.	- **Experimental group:** Li-ESWT.<br>- **Control group:** Placebo.	- **Groups by energy density:** 0.09 mJ/mm^2 and 0.25 mJ/mm^2.<br>- **Groups by number of pulses per treatment:** 14.400-20.000, 18.000 and 36.000 pulses.	**- 5/12 RCT:** 2 times/week for 3 weeks + 3 weeks rest + 2 sessions/week for another 3 weeks.<br>**- 3/12 RCT:** 4 sessions/week.<br>**- 3/12 RCT:** 2 sessions/week for 6 weeks.	**- Erectile function** (IIEF-EF).<br>- **Erection hardness score** (EHS).<br>**- Sexual Health Inventory for Men** (SHIM).<br>- **Patient satisfaction.**<br>**- Treatment safety.**	1, 3, 6 and 12 months.	- **IIEF-EF:** A significant increase was observed in the experimental group after 1 month (MD = -2.92; 95% CI, -3.17 to -2.67; Z = 22.87; p = 0.000) compared to baseline, to a greater extent than placebo (MD= -0.99; 95% CI, -1.31 to -0.67; Z = 6.07; p = 0.000). At 3-6 months, the difference was still significantly greater than baseline (MD= -2.78; 95% CI, -3.17 to -2.67; Z = 22.87; p = 0.000) in the experimental group. 1/12 RCT compared the efficacy of placebo at 3-6 months to baseline (MD = -9.14; 95% CI, -11.28 to -7.01). The comparison between Li-ESWT and placebo at 1 month favors the active treatment (MD = 2.53; 95% CI, 2.11 to 2.95; Z = 11.91; p = 0.000).

**Liu et al. 2022 [35]**	11 RCT and 814 participants.	- **Experimental group:** Li-ESWT.<br>- **Control group:** Li-ESWT without energy transmission.	- **Groups by energy density:** 0.09 mJ/mm^2 and 0.25 mJ/mm^2.<br>- **Groups by number of pulses per treatment:** 15.000 and 18.000 pulses.	- 1 session/week for 4 (1/11 RCT), 5 (1/11 RCT) and 10 weeks (1/11 RCT).<br>- 2 sessions/week for 2 (1/11 RCT), 3 (1/11 RCT), 6 (4/11 RCT) and 12 weeks (1/11 RCT).	- **Erectile function** (IIEF-EF).<br>- **Erection hardness score** (EHS).	7 weeks and 1, 3, 6, 9 and 12 months.	- **IIEF-EF:** Significant increase in mean IIEF-EF score in the experimental group [(MD = 2.77; 95% CI (1.74, 3.79); I^2 = 66%; p < 0.001)] compared to the control group. Clearly elevated changes in IIEF-EF score [(MD = 3.75; 95% CI (3.15, 4.35); p < 0.001)] in the experimental group.<br>- **EHS:** 7/11 RCT → Clearly elevated EHS scores after Li-ESWT (OR: 9.37; 95% CI [5.65, 15.52]; I^2=61%, p < 0.001).
**Sokolakis 2019 [37]**	10 RCT and 872 participants.	- **Experimental group:** Li-ESWT.<br>- **Control group:** simulated control therapy.	- **Groups by energy density:** ranged from 0.05-0.25 mJ/mm^2. Most studies used 0,09 mJ/mm^2.<br>- **Groups by number of pulses per treatment:** ranged from 600 to 5.000 pulses. Most studies administered 1.500 pulses/treatment (total =3.000-60.000 pulses).	- Sessions ranging from 1 to a maximum of 5 sessions/week (typically 1 or 2 sessions/week) for 1-9 weeks.	- **Erectile function** (IIEF-EF).<br>- **Erection hardness score** (EHS).<br>- **Peak systolic velocity** (PSV).<br>- **Sexual encounter profile** (SEP).<br>- **Global assessment questionnaire** (GAQ).<br>- **Maximum penile circumferential change and clinical global impression of change** (CGIC).	1, 3, 6 and 12 months.	- **IIEF-EF:** Significantly greater change in IIEF-EF score in the experimental group from baseline (MD:3.97; 95% CI [2.09-5.84]; p < 0.0001), as well as at follow-up (MD: 3.71; 95% CI [0.29-7.14]; p=0.03) compared to the sham-controlled group. The percentage of patients who achieved the MCID on IIEF-EF was significantly higher in the Li-ESWT group than in the control group (OR: 8.54; 95% CI [2.64, 27.63]; p < 0.0003).<br>- **EHS:** A higher percentage of patients in the experimental group achieved an EHS ≥ 3 at follow-up compared to the control group (OR 4.35; 95% CI [1.82-10.37]; p=0.0009).<br>- **PSV:** Li-ESWT significantly increases PSV from baseline compared to sham therapy (MD: 4.12; 95% CI [2.30-5.94]; p≤ 0.00001) and reaches higher levels at follow-up (MD: 4.48; 95% CI [2.60-6.35]; p<0.00001).
